# Isolated Cervical Dystonia: Management and Barriers to Care

**DOI:** 10.3389/fneur.2020.591418

**Published:** 2020-11-27

**Authors:** Melanie Leigh Supnet, Patrick Acuna, Samuel J. Carr, Jan Kristoper de Guzman, Xena Al Qahtani, Trisha Multhaupt-Buell, Taylor Francoeur, Gabrielle E. Aldykiewicz, Priyanka R. Alluri, Lindsey Campion, Lisa Paul, Laurie Ozelius, Ellen B. Penney, Christopher D. Stephen, Marisela Dy-Hollins, Nutan Sharma

**Affiliations:** ^1^Department of Neurology, Massachusetts General Hospital and Harvard Medical School, Boston, MA, United States; ^2^Department of Neurology, Jose R. Reyes Memorial Medical Center, Manila, Philippines

**Keywords:** cervical dystonia, movement disorders, treatment, barriers to care, clinical features

## Abstract

**Background:** Cervical dystonia (CD) is a rare disorder, and health care providers might be unfamiliar with its presentation, thus leading to delay in the initial diagnosis. The lack of awareness displays the need to highlight the clinical features and treatment in cervical dystonia. In our cohort, we have identified an earlier age of onset in men, despite an overall preponderance of affected women.

**Objective:** We aim to identify the prevalence, age of onset, spread, and treatment modalities of CD in the population. We also highlight the barriers which patients encounter related to diagnosis, follow-up, and treatment.

**Methods:** We reviewed 149 CD patients who attended specialized Dystonia Clinics over a 14-year period. Dystonia severity was rated using the Burke-Fahn-Marsden (BFM), Tsui, and Toronto Western Spasmodic Torticollis Rating Scales (TWSTRS). Mood and quality of life were assessed using Beck Depression Inventory (BDI), Beck Anxiety Inventory (BAI), and 36-Item Short Form Health Survey (SF-36).

**Results:** CD patients were majority White (91.3%) and more commonly female (75.8%). Men had an earlier median age of onset, 40.5 years (*p* = 0.044). BAI revealed a mean score of 7.2 (±6.4, *n* = 50) indicating minimal anxiety while BDI revealed a mean score of 7.30 (±7.6, *n* = 50) indicating minimal depression. The only SF-36 subscales associated with CD severity were physical functioning (*p* = 0.040) pain (*p* = 0.008) and general health (*p* = 0.014).

**Conclusion:** There appear to be gender differences in both the prevalence and age of onset of the disease. There was a 3-fold higher incidence in women than in men. CD patients of both sexes experience barriers to care, which can be reflected in their quality of life and time-to-diagnosis. In addition, males were less likely to experience an objective benefit with botulinum toxin treatment and more likely to discontinue care. Greater awareness of CD by health care providers is important to reduce the time-to-diagnosis.

## Introduction

Isolated cervical dystonia (CD) is a common subtype of dystonia which involves abnormal neck movement, is frequently associated with pain, can be debilitating (1, 2), and has an estimated prevalence of 3–9 per 100,000 (3–6). Isolated CD is a rare condition, with the greatest incidence in White females and a mean age of onset between 45 and 50 years of age, and where the majority of patients do not have spread to other body parts (5, 7–10). There is a growing body of literature on the non-motor features of CD, including executive dysfunction, depression, anxiety, pain, and increased disability, decreased quality of life (7, 11–13). Those with CD display a high rate of depression or anxiety that has not previously correlated with dystonia severity (14). The main treatments in cervical dystonia are: botulinum toxin injections alone; botulinum toxin injections in combination with oral medication; and for refractory cases, deep brain stimulation (DBS) (7, 15).

Since cervical dystonia is a relatively rare disease, many physicians have little exposure while in training or clinical practice, which may lead to a delay in diagnosis and treatment. Delay in diagnosis of CD ranges from 3.75 to 6.8 years, with subjects having seen multiple providers prior to diagnosis (16–18). The observed delay to diagnosis has been attributed, in part, to a lack of awareness within the healthcare profession (17). As CD can be treated, better recognition of the disorder would likely provide faster diagnosis and symptom relief to more individuals (16).

To date, there has been limited data on barriers to care for those with cervical dystonia. One study, involving 70 CD patients at an academic movement disorders clinic in the United States, examined the treatment experiences and reasons for discontinued care (19). Thirty percent of patients discontinued their care, and the most cited reasons were a suboptimal response to or side effects from botulinum toxin, high cost of treatment, and excessive travel burden.

Here, we report on our findings in a CD cohort diagnosed and treated over 14 years at two large academic medical centers. We also report on unique features in our cohort, namely a marked gender difference in age of onset and the lack of significantly higher comorbid depression or anxiety than that found in the general population. This data adds to the existing literature on CD and points to greater heterogeneity in affected individuals than previously reported.

## Methodology

### Subject Participation

Subjects were recruited prospectively from the Dystonia Clinics at Massachusetts General Hospital and Brigham and Women's Hospital. We obtained coded data from 149 consecutive participants who were diagnosed with isolated CD, with or without involvement of other body regions and enrolled in the Dystonia Partners Research Bank (DPRB) between January 2005 and December 2019. Twenty-five of the 149 subjects had previously enrolled in an observational study on dystonia and had additional motor scale scores. When available, we included prior motor scale scores for those subjects. All DPRB participants gave written informed consent for their data to be used in a natural history analysis. Our data analysis protocol was reviewed and approved by the Mass General Brigham Human Research Committee.

Diagnosis and confirmation that the subjects had isolated cervical dystonia, including a review of previous medication exposure, were determined according to published criteria (20, 21) by a neurologist trained in movement disorders (NS, MD, EP, CS). We assessed demographics, including time from symptom onset to diagnosis, family history, medical history, and prescribed medications. Subjects were assessed using a standardized movement disorder examination as described previously (22). Dystonia severity was rated in all patients using the Burke-Fahn-Marsden Motor Rating Scale (BFMMS) and in a subset of subjects with the Tsui Dystonia Scale and the Toronto Western Spasmodic Torticollis Rating Scale (TWSTRS). Follow-up examinations were completed in 98 participants, which all occurred at least 9 months after the first examination and around the time of medical appointments.

### Molecular Analysis

DNA was extracted from white blood cells using the Purgene procedure (Gentra Systems, Minneapolis, MN). The samples were screened for presence of the TOR1A (DYT1) GAG deletion by PCR amplification as previously described (21).

### Socio-Economic Factors

Insurance status was provided through medical record review and coded as either private or public, with the latter consisting of Medicare and Medicaid health insurance plans. Zip code, obtained at enrollment and/or medical record review, was used to calculate median household income using 2017 United States Government Census data [ref “American Fact Finder”]. The distance from the clinic was calculated by using the subject's address and the address of the clinic they attended.

### Mood and Quality of Life Assessment

We obtained depressive and anxiety symptoms from the Beck Depression Inventory, 2nd edition (BDI) (23), and the Beck Anxiety Inventory (BAI) (24). The impact of CD on Health-Related Quality of Life (HRQoL) was assessed using the 36-Item Short Form Health Survey (SF-36) (25). Fifty consecutive patients completed the BAI, BDI, and 49 completed the SF-36, which were given to participants and then returned by mail.

### Statistical Analysis

We performed descriptive statistics. Categorical variables were compared with χ^2^ or Fisher's exact test. For continuous variables we used two-sample *t*-tests, and for highly skewed data the Wilcoxon rank sum test. We used Spearman's correlation coefficient to assess correlation in continuous variables. Changes in motor scores from baseline to their final scores were assessed using Wilcoxon signed-rank test for matched pairs. A one-sample *t*-test was used to compare the differences between the cohort's SF-36 subscores/general health and the corresponding Ware's normative values (26). Statistical significance was designated by two-tailed *p* < 0.05. Data was analyzed using STATA 15.1 (College Station, TX).

## Results

Data from a total of 149 subjects was used. Of these, 36 (24.2%) were male and 113 (75.8%) were female, as shown in Table 1. When analyzed by gender, the median age of onset of symptoms in the male cohort (*n* = 36) is 40.5 years (IQR = 27.5–48.5), and the median age of onset in the female cohort (*n* = 113) is 45 years (IQR = 36.0–55.0). Males had an earlier age of onset of symptoms compared to females (*p* = 0.044). Males also had an earlier age of diagnosis, 53.5 years (IQR = 47–60.5 years), when compared to females, 58 years (IQR = 49–66 years) (*p* = 0.043). Gender differences with respect to time-to-diagnosis were not significant (*p* = 0.874).

**Table 1:** Clinical and demographic characteristics.

	**Total (*n* = 149)**	**Male (*n* = 36)**	**Female (*n* = 113)**	***p*-value**
Age of onset, Median (IQR)	45 (34–53)	40.5 (27.5–48.5)	45 (36–55)	0.044
Age of diagnosis, Median (IQR)	57 (48–65)	53.5 (47–60.5)	58 (49–66)	0.043
Time to diagnosis, Median (IQR)	10 (4–18)	10 (5–18)	10 (3–18)	0.874
**Ethnicity**				0.516
White non-hispanic	136 (91.3%)	32 (88.9%)	104 (92.0%)	
Other	13 (8.7%)	4 (11.1%)	9 (8%)	
Family history^*^				0.191
Negative	69 (50.7%)	13 (40.6%)	56 (53.8%)	
Positive	67 (49.3%)	19 (59.4%)	48 (46.2%)	
Dystonia spread^**^				0.578
Isolated	75 (76.5%)	17 (77.3%)	58 (76.3%)	
Spread	23 (23.5%)	5 (22.7%)	18 (23.7%)	
**TREATMENT**				
**Botox**				0.049
Continued	95 (63.8%)	18 (50%)	77 (68.1%)	
Stopped	54 (36.2%)	18 (50%)	36 (31.9%)	
**Dystonia medications**				0.499
Yes	61 (40.9%)	13 (36.1%)	48 (42.5%)	
No	88 (59.1%)	23 (63.9%)	65 (57.5%)	
**DBS**				0.618
No	141 (94.6%)	34 (94.4%)	107 (94.7%)	
Yes	8 (5.4%)	2 (5.6%)	6 (5.3%)	
**Antidepressant medications**				0.316
Yes	25 (16.8%)	8 (22.2%)	17 (15.0%)	
No	124 (83.2%)	28 (77.8%)	96 (85.0%)	
**SOCIODEMOGRAPHICS**				
Median household income, Median (IQR)	89,440 (65,476–110,025)	90,285 (69,402–122,393)	88, 993 (65,348–106,332)	0.310
Miles from clinic, Median (IQR)	35.65 (13.9–56.3)	41.35 (13.6–58.6)	34.15 (14.3–56.1)	0.794
**Insurance**				0.010
Private	93 (62.4%)	29 (80.6%)	64 (56.6%)	
Public	56 (37.6%)	7 (19.4%)	49(43.4%)	

* *Family History analyzed for positive/negative subjects only (136/149). Thirteen subjects had an unknown/possible history which researchers could not analyze effectively*.

** *Dystonia spread available for subjects with >1 visit and followed for at least 0.75 years (98/149)*.

When compared to the overall race/ethnicity and gender data in the outpatient specialty care population at Massachusetts General Hospital (MGH), our subjects are more likely to be White (91.3% in this cohort vs. 76% in the MGH outpatient specialty care population), not Hispanic or Latino (96.64% in this cohort vs. 91.2% in the MGH outpatient specialty care population) and female (75.8% in this cohort vs. 51.8% in the MGH outpatient specialty care population) (27).

For 98 of the subjects, we had at least one follow-up study visit, obtained at least 9 months after the enrollment visit. In this subset, 76.5% of subjects (75/98) had dystonia restricted to the neck. In 23.5% of subjects with follow-up (23/98), dystonia spread from the neck to adjacent body regions, resulting in segmental dystonia (Table 1). One subject developed multi-focal dystonia, affecting both hands as well as upper and lower facial muscles; one subject developed generalized dystonia; two individuals first developed dystonia in an adjacent body region (spasmodic dysphonia or lower face) with subsequent extension to the neck. Using a Fisher exact test, there was no significant correlation between dystonia spread and family history of dystonia, tremor, Parkinson's Disease, and/or myoclonus (*p* = 0.729).

### Family History

Analysis of our cohort's first and second-degree relatives revealed that 47.0% (70/149) had a positive family history of movement disorders (dystonia, tremor, Parkinson's Disease, and/or myoclonus), 45.0% (67/149) had a negative family history, and 8.0% had a possible/unknown family history (12/149). The 67 subjects with a positive family history of a movement disorder were tested for presence of the DYT1 mutation as this has been reported to result in cervical dystonia in rare cases and is a common cause of familial disease (28).

### Socioeconomics

All subjects were covered by a health insurance plan, with 62.4% having private insurance (93/149) and 37.6% having publicly funded insurance (56/149, Medicare, or Medicaid). A higher proportion of males had private insurance compared to females (*p* = 0.01). In the Commonwealth of Massachusetts, the Center for Health Information and Analysis (CHIA) conducted a Health Insurance Survey in 2019 and found that 92% of residents had health insurance coverage for the entire year. Of those with insurance, 64.4% had private insurance and 31.2% had publicly funded insurance (Medicare or Medicaid). Based on the chi squared goodness of fit model, there is no statistically significant difference (*p* = 0.09) between our insurance rates in our cohort and that of the state. An additional 3.1% of state residents were covered by private, non-group coverage plans and none of these residents were represented in our cohort. Median household income was $89,440 (IQR = 65,476–110,025). Median miles from the clinic was 35.65 (IQR 13.9–56.3). In our cohort, the median for time to diagnosis (age at diagnosis—the age at onset) was 10 years (IQR = 4–8). We found there was no correlation between time to diagnosis and race, public vs. private insurance, miles to the clinic, nor median household income.

### Dystonia Rating Scale Scores

At baseline exam, data from 147 subjects revealed a mean score of 4.6 on the Burke Fahn Marsden (SD = 2.2), 144 subjects had a mean score of 6.1 (SD = 2.2) on the Tsui, and 140 subjects had a mean score of 16.9 (SD = 4.9) on the TWSTRS severity scale. At the most recent exam, data from 102 subjects revealed a mean score of 4.5 on the Burke-Fahn-Marsden (SD = 5.1), a mean score of 5.7 (SD = 2.5) on the Tsui, and a mean score of 14.8 (SD = 6.3) on the TWSTRS severity scale. There were no differences in dystonia severity scores between the sexes when we take into account 95 subjects with all three movement scores on baseline exam and at most recent exam (Table 2).

**Table 2:** Motor rating scale scores.

	**Total (*n* = 95)**	**Male (*n* = 20)**	**Female (*n* = 75)**	***p*-value**
BFM V0, Mean (SD)	4.68 (1.87)	4.9 (1.80)	4.63 (1.90)	0.556
BFM VF, Mean (SD)	3.89 (2.21)	4.53 (1.87)	3.72 (2.27)	0.112
TSUI V0, Median (IQR)	6 (5–8)	6 (6–8)	6 (5–8)	0.596
TSUI VF, Median (IQR)	6 (4–7)	6.5 (4.5–7.5)	6 (4–7)	0.224
TWSTRS V0, Median (IQR)	18 (16–20)	19 (17.5–21)	18 (15–20)	0.374
TWSTRS VF, Median (IQR)	15 (10–20)	16 (10.5–20.5)	15 (10–19)	0.654

### Mood and Quality of Life

Of the 50 subjects who completed the non-motor scales, 6 (12.0%) were male and 44 (88.0%) were female. BAI revealed a mean anxiety score of 7.2 (±6.4), indicating minimal anxiety (Table 3). The distribution of BAI scores revealed 68% (34/50) with minimal anxiety, 16% (8/50) with mild anxiety, and 16% (8/50) with moderate anxiety. BDI revealed a mean depression score of 7.30 (±7.6, *n* = 50), indicating minimal depression. The distribution of BDI scores revealed 86% (43/50) with minimal depression, 6% (3/50) with mild depression, 6% (3/50) with moderate depression, and 2% (1/50) with severe depression. Those with severe scores on the BDI were referred to his/her clinical neurologist and offered resources for support. There was no correlation between BAI or BDI with CD severity at baseline or the most recent exam based on TWSTRS (severity), BFM, or TSUI scores. The only SF-36 subscales to be associated with CD severity were physical functioning (*p* = 0.040) pain (*p* = 0.008) and general health (*p* = 0.014) at baseline using the TWSTRS severity scale. Upon further examination, the participants who completed the BDI and BAI scales had statistically better BFM, TWSTRS and TSUI movement scores at their most recent exam than those who did not complete the mood scales (BFM *p* = 0.03, TWSTRS *p* = 0.02, TSUI *p* = 0.03). Furthermore, we found that patients who completed the BDI and BAI were more likely to appear for their regular botulinum toxin injection visits (*p* < 0.0001).

**Table 3:** Non-motor rating scale scores.

	**Total (*n* = 50)**	**Male (*n* = 6)**	**Female (*n* = 44)**
Beck Anxiety Inventory (*n* = 50)	7.2 ± 6.4	2.86 ± 3.29	7.88 ± 6.58
Beck Depression Index II (*n* = 50)	7.3 ± 7.6	5 ± 2.94	7.67 ± 8.05
**SF-36**	**Total (*n* = 49)**	**Male (*n* = 6)**	**Female (*n* = 43)**
Physical functioning	69.3 ± 25.4	82.14 ± 14.96	67.14 ± 26.30
Role limitations due to physical health	54.3 ± 44.1	78.57 ± 36.60	50.30± 44.39
Role limitations due to emotional problems	72.4 ± 38.1	100 ± 0	67.86 ± 39.37
Energy/fatigue	54.5 ± 20.9	68.57 ± 14.06	52.11 ± 21.03
Emotional well-being	75.6 ± 17.8	82.85 ± 12.59	74.43 ± 18.35
Social functioning	74.7 ± 21.6	83.93 ± 21.30	73.21 ± 21.49
Pain	63.9 ± 20.1	67.86 ± 15.64	63.21 ± 20.82
General health	66.0 ± 22.2	67.14 ± 17.99	65.83 ± 23.03

*All patients were receiving botulinum toxin injection*.

*27 patients were on oral medications*.

*9 patients were on antidepressants*.

### Treatment

We closed the study on December 31, 2019. At that time point, most subjects were being treated regularly with botulinum toxin injections using electromyographic guidance (75%, 112/149). Many who underwent botulinum toxin injections were doing so quarterly (46.97%, 70/149) with others either choosing to undergo injections less frequently (16.78%, 25/149) or changing providers for their treatment due to insurance requirements or travel distance (11.41%, 17/149). Of the 37 subjects who did not get regular treatment with botulinum toxin, 12 (8.05%) had never tried it or felt it did not work, and 17 (11.41%) found that the costs and discomforts of treatment with botulinum toxin were not worth the benefit. Eight of the 37 (5.37%) had either died or faced other serious medical conditions (not associated with our study or their dystonia) that impeded access to regular botulinum toxin injections. Before these circumstances arose for the eight subjects, four had been receiving quarterly injections, one was stretching their injections, another had switched providers but continued treatment, and two had stopped their injections altogether. Women were more likely to continue receiving botulinum toxin than men (p = 0.049). Based on the Wilcoxon signed-rank test for matched pairs, there was a statistically significant difference in baseline vs. final movement scores (BFM-*p* = 0.0001, TSUI-*p* = 0.0027, TWSTRS-*p* = 0.0009, *n* = 75) in women who received botulinum toxin injections but not in men, indicating a measurable benefit of long-term treatment for women.

In addition to interval treatment with botulinum toxin, daily oral pharmacologic treatment was used in some subjects to treat dystonia or depression (Table 1). While 59.1% (88/149) were not on any medications to alleviate dystonia, 19.5% (29/149) were treated with benzodiazepines, 4.7% (7/149) were treated with skeletal muscle relaxants, 1.3% (2/149) were treated with propranolol to alleviate a concomitant tremor, 0.7% (1/149) was on primidone to alleviate a tremor, 0.67% (1/149) was on an alpha-adrenergic agonist, and 11.4% (17/149) were on two or more medications to alleviate dystonia. In addition, 16.8% (25/149) were under treatment for depression. In those treated for depression, 56.0% (14/25) were prescribed selective serotonin reuptake inhibitors, 20.0% (5/25) were prescribed selective norepinephrine reuptake inhibitors, 20.0% (5/25) were prescribed tricyclics, and lastly one subject was prescribed a dopamine reuptake inhibitor 4.0% (1/25).

Furthermore, 5.4% (8/149) were treated with deep brain stimulation (DBS) due to minimal or suboptimal response to treatment with botulinum toxin injections and oral medications. The use of DBS treatment was not significantly correlated with the severity of a subject's TWSTRS severity score at baseline or with an incidence of dystonia spread. In all eight subjects, the DBS targeted the globus pallidus interna (GPi). Pre and post DBS scores were available for four subjects, whose relief ranged from 2 to 4 points on the TWSTRS motor scale. Albeit a small sample size, six of the subjects no longer receive botulinum toxin injections and have found substantial relief of their symptoms with DBS alone.

## Discussion

In this cohort of 149 patients diagnosed to have isolated dystonia, there appear to be gender differences in both the prevalence and age of onset of the disease. We found a 3-fold higher incidence in women than in men, consistent with previous reports (29, 30). Interestingly, we found a significant difference between genders in the mean age of onset, with men displaying symptoms nearly 7 years earlier than women. While our overall age of onset does align with other smaller studies, indicating that CD is an adult-onset disease most commonly occurring around ages 40–45, we believe that this is the first report to identify a significantly younger age of onset in affected men. It is unclear whether these sex differences are the result of our relatively small sample size or indicative of a genetic predisposition/vulnerability that is specific to men with cervical dystonia. However, we did not see a sex difference in those who had a family history of movement disorders, arguing against a clear genetic predisposition to the development of CD in men.

This study is one of the first to look into barriers to care within cervical dystonia. We identified a significant range of values (nearly 50 years) and distribution (tailing to the right) in our cohort's median time to diagnosis 10 years. This suggests a disparity in access to healthcare and/or access to physicians familiar with cervical dystonia that is exaggerated in a portion of the cohort. The cohort's median time to diagnosis, 10 years (IQR = 4–18), was nearly double that reported in an Australian study of 50 subjects with CD and three times that reported in a US study (16, 31). We did not find a significant interaction between time to diagnosis and race/ethnicity, insurance status, median household income, or miles from the clinic. Upon further investigation on the 10–15% at the extreme end of the distribution spectrum, there does not seem to be any statistically noticeable pattern that distinguishes them from the larger cohort based on the data we collected, including race, public vs. private insurance, miles to the clinic, and median household income.

In those who received a diagnosis of cervical dystonia and had access to treatment, ~11% felt that botulinum toxin was not worth the pain or cost and 8% had no benefit or never tried it. Botulinum toxin is one of the main treatment strategies for CD, and the majority of this cohort was receiving injections regularly (47% quarterly, 17% stretching their injections longer) and finding measurable benefits from the treatment. There was a significant reduction in all three measures of dystonia severity (BFM, Tsui, and TWSTRS) from baseline to the most recent exam in women receiving botulinum toxin injections but not in the men. Similar to the sex difference we found in age of onset, this finding may be due to a small sample of male participants or indicative of sex-based differences in response to treatment. In our sample of 149 subjects, 5.4% opted for treatment with deep brain stimulation (DBS). While case series have shown its effectiveness in CD, a multi-center prospective study analyzing its efficacy, including patient-reported outcome measures, may help determine which patients would most benefit (32).

For those who discontinued their care at the clinic, 57.9% did not cite a complete lack of benefit from botulinum toxin but either found that there was an insufficient pharma-economic benefit or an excessive travel burden involved. These results are in line with previous reports, which cited that reasons for discontinuation of care include high cost, suboptimal response to or side effects from botulinum toxin, and excessive travel burden (7, 17). Males were more likely to discontinue their botulinum toxin care, which may be associated with the lack of a significant reduction in their objective measures of dystonia severity. Although the current study attempted to further understand barriers to care by measuring socio-economic status (SES) through city median household income and measuring miles from the clinic, there was no direct correlation with care outcomes.

Previous studies have demonstrated the coexistence of severe depression and anxiety with isolated cervical dystonia (14). In contrast, our findings indicate that although depression and anxiety are present among these patients, their scores fall into a minimal range. Additionally, our average BAI and BDI scores were slightly lower than those presented in a cohort of 255 CD subjects (12). While the BDI and BAI have not been validated for dystonia, they have been used in previous studies of dystonia cohorts (2, 12). All of the patients who completed the non-motor scales were receiving regular botulinum toxin injections. Evidence suggests that the severity of depressive symptoms decreases with the successful use of botulinum toxin therapy for CD (33). This may explain the lower rate of acute, untreated depression and anxiety in our cohort. However, additional studies to both validate the use of the BDI and BAI in dystonia subjects, and determine appropriate cutoff scores to identify mood disorders are required.

In our cohort, the SF-36 subscales that showed a significant correlation with CD severity (as measured by the TWSTRS) were physical functioning, pain, and general health. To our knowledge, this is the first report to demonstrate a correlation between the TWSTRS severity score, an objective measure of motor dysfunction, and patient-reported physical function, pain and general health perception. Taken together, our participant-reported data indicate that CD motor severity correlates with reduced quality of life and daily physical function, but not with underlying depression or anxiety. SF-36 scores for the patients with CD were significantly lower (worse) than normative values in the US population in the domains of physical functioning, role limitations due to physical health, social functioning, and pain (26, 34). These findings suggest that the highest burden from CD on quality of life is physical and social, rather than mental or emotional. A subsequent study is needed to better understand the psychiatric profile of cervical dystonia, and this should include asking subjects to complete the questionnaires during every stage of the study to control for confounding variables such as treatment effectiveness.

In summary, this study presents a cohort of CD patients who have a significantly younger age of onset in males, and a high prevalence of women and White non-Hispanic individuals. Our study found no significant psychiatric associations with mood disorders, such as anxiety or depression, which have been reported in other studies of CD patients. Additionally, our findings suggest that the CD population does experience barriers to care, which can be reflected in their treatment dropout rates and time-to-diagnosis, the latter of which could average well above 10 years. A future study on the natural history of cervical dystonia and barriers to care should include using more comprehensive indicators of socio-economic status, such as a subject's self-reported household income, educational status, and travel time rather than the distance from the clinic. Additionally, future research should be conducted in a larger, more racial/ethnic diverse cohort.

## Limitations

Our cohort is overwhelmingly White and non-Hispanic/Latino. This could be interpreted in multiple ways, including: an artefactual finding due to relatively small sample size; an accurate indicator of the predominance of cervical dystonia in White non-Hispanic populations; or the result of reporting from a highly specialized neurologic clinic that under-serves minorities, which is reflected in their lack of participation in research at academic healthcare centers. A better understanding of the epidemiology and barriers to care within the cervical dystonia population is needed, but these results do align with a multi-ethnic American study that cited a higher prevalence of CD in White individuals (5).

Regarding the etiology of CD in this cohort, all subjects with a positive family history of movement disorder were screened and found to be negative for the DYT1 mutation. While genetic causes of isolated CD are thought to be rare, it is possible that some members of our cohort carry other dystonia-causing genes, such as DYT25 or DYT6, for which we did not screen.

The findings presented on mood and quality of life suggest that the highest burden from CD on quality of life is physical and social, rather than mental or emotional. However, these findings may be confounded by selection bias, because only one-third of the cohort completed psychiatric and QoL questionnaires, and these forms were dispensed at follow-up visits to subjects who continued with treatment. Furthermore, those who are psychiatrically well may be more likely to actively participate in research than those with more mental/emotional burden from CD or other diseases (25). Our study is limited to the patients who were able to start and continue treatment at our clinic, and specifically those who were willing and able to participate in research before or after their medical appointments. Additionally, not all subjects were tracked on the same interval schedule due to their personal availability. A subsequent study is needed to better understand the psychiatric profile of cervical dystonia, and this should include asking subjects to complete the questionnaires during every stage of the study to control for confounding variables such as treatment effectiveness.

## Data Availability Statement

The raw data supporting the conclusions of this article will be made available by the authors, without undue reservation.

## Ethics Statement

The studies involving human participants were reviewed and approved by Mass General Brigham Human Research Committee. The patients/participants provided their written informed consent to participate in this study.

## Author Contributions

MS, PA, SC, MD-H, and NS: conception, organization, and execution of research project, design, execution, and review and critique of statistical analysis, and writing of the first draft and review and critique of the manuscript. JK, XA, TF, PA, LC, LP, and LO: execution of the research project and review and critique of the statistical analysis and manuscript. TM-B, EP, and CS: conception, organization, and execution of the research project and review and critique of the statistical analysis and manuscript. GA: organization and execution of the research project and review and critique of the statistical analysis and manuscript. All authors contributed to the article and approved the submitted version.

## Conflict of Interest

The authors declare that the research was conducted in the absence of any commercial or financial relationships that could be construed as a potential conflict of interest.
